# Adeps Lanae—An Ideal Ointment Base

**Published:** 1896-10

**Authors:** Bernard Wolff

**Affiliations:** Atlanta, Ga.; Lecturer on Dermatology and Syphilis, Southern Medical College


### ADEPS LANAE—AN IDEAL OINTMENT BASE.

By BERNARD WOLFF, M.D., Atlanta, Ga.

Lecturer on Dermatology and Syphilis, Southern Medical College.

The medical public differs not at all from the lay public in its reluctance to avail itself of a new thing when it is apparently satisfied with the old. This is especially true of the ointment bases in which remedies are used by the general practitioner for the relief of disease in the skin. For years lard in its various combinations reigned supreme, until the powerful supporters of vaseline broke its sway and put their favorite in the ascendency.

The claims of vaseline to supremacy as a vehicle for remedies used in the form of salves have been of late years so energetically assailed from so many points at once that it has decidedly lost caste. At present, vaseline or what is the same thing , petrolatum, has been unquestionably relegated to a back seat to give place to lanolin, agnine, adeps lanae, etc. It would be no matter of surprise to see it playing the minor parts of an adjuvant or even a lubricant for instruments, or as a rival to the once valued bear's grease, to impart that peculiarly unctuous lustre to the hair which is so highly prized by a certain class of people. But the general practitioner clings tenaciously to vaseline and seems loth to desert it even though the advantages of a purified animal fat which does not become rancid, can not fail to impress him. The difficulty is to get him to try it. The "old-time Bible is good enough for me," said a lot of old fogies some years ago, and would have set at naught the years of patient toil of the revisers. Such sentiments appeal to the gallery, but a moment's reflection will show how destructive they are to progress.

By far the most elegant ointment base now in use is adeps lanae. It is a definite chemical compound and does not undergo retrograde change and become rancid, or acquire a disagreeable odor. It is neutral in reaction and can be used as a vehicle for any medicament. Its miscibility with water, of which it can take up 300 per cent. without losing its salve-like consistency, makes it a very easy matter to reduce it to the desired consistency. It melts at the temperature of the body and possesses, in a remarkable degree, the power of being absorbed through the unbroken integument, a quality to which vaseline lays a spurious claim.

Ointments made from it have an incomparable smoothness, and unctuousness, and do not adhere to the skin in such a way as to be removed with difficulty.

Adeps lanae used alone is an excellent emollient, softening the skin and rendering it smooth and pliant. It has slightly curative powers when applied to irritated surfaces, such as dermatitis from sunburn.

Its principal use is as a menstruum for incorporation of various remedies for topical application in the form of an ointment. I first used adeps lanae in Unna's clinic at Hamburg, in 1893, where it had supplanted every other form of ointment base.

I subjoin a few formulæ which I have found of value in my dermatological practice.

COLD CREAM.

℞ Adeps lanae, N. W. K ........ 10.0
Vaseline alb ........ 15.0
Aq. destil ........ 20.0
Zinc. oxid ........ 10.0
M.—Fnt. ung.

BLAND SALVE.

℞ Adeps lanae, N. W. K ........ 10.0

Ol. amygdal ........ 5.0

Aq. aurant. flor ........ 15.0

M.—Fnt. ung.

Or

℞ Adeps lanae, N. W. K. ........ 15.0
Vaseline ........ 15.0
Potas. carbonat ........ 1.0
Aq. rosæ ........ 20.0
M.—Fnt. ung.

PSORIASIS, CHRONIC ECZEMA, ETC.

℞ Adeps lanae, N. W. K., } aa ........ 25.0

Vaseline }

Chrysarobin ........ 3.0

M.—

For the same.

℞ Chrysarobin ........ 5.0

Ol. cadini ........ 10.0

Sulph. præcip ........ 5.0

Ol. olivar ........ 10.0

Adeps lanae, N. W. K ........ 20.0

M.—Fnt. ung.

ECZEMA OF THE LIDS.

℞ Hydrarg. oxid. flav........ 0.1
Adeps lanae, N. W. K........ 2.5
Ol. amygdal........ 2.5
Aq. rosæ........ 5.0
M.—Fnt. ung.

"BLEACHING" SALVE.

℞ Aluminis........ 1.0

Hydrogen dioxid........ 10.0

Adeps lanae, N. W. K. } aa........ 5.0

Ol. amygdal }

M.—Fnt. ung.

COOLING SALVE.

℞ Adeps lanae, N. W. K........ 15.0
Lanolin........ 10.0
Aq. destil........ 25.0
M.—Fnt. ung.

FOR ECZEMA SEBORRHEIC.

℞ Zinc oxid........ 2.0
Acid carbol........ 1.0
Sulph. præcip........ 2.0
Adeps lanae, N. W. K........ 20.0
Aq. rosæ........ 5.0
M.—Fnt. ung.

ROSACEA.

℞ Resorcin........ 2.0
Acid salicyl........ 0.5
Sulph. præcip........ 1.0
Adeps lanae, N. W. K........ 10.0
Aq. rosæ........ 15.0
Vaselin........ 5.0
M.—Fnt. ung.

---

A CURIOUS effect of the X-rays has been noticed by Dr. M. J. Stern in his experiments at the Philadelphia Polyclinic Hospital; namely, a reddening and finally a bronzing of the skin of the hands from constant exposure to the radiations in handling the apparatus. The general appearance and course is that of slow sun-burning; and the physiologic mechanism is probably similar.—*Exchange.*

